# Female Fingerprint: Sex-Specific Aspects in Patients With Atrial Fibrillation and Heart Failure

**DOI:** 10.1007/s11897-026-00790-9

**Published:** 2026-11-06

**Authors:** Esther Roth, Laura Rottner

**Affiliations:** 1 University Heart and Vascular Center Frankfurt, Goethe University Frankfurt, Frankfurt am Main, Germany; 2 University Heart and Vascular Center Frankfurt, Theodor-Stern Kai 7, 60596 Frankfurt am Main, Germany

**Keywords:** Gender-sensitive cardiovasculare care, Female phenotype, Heart failure, Atrial fibrillation, Women’s heart health center

## Abstract

**Purpose of Review:**

Atrial fibrillation (AF) and heart failure (HF) frequently coexist and represent a major source of cardiovascular morbidity and mortality. This review summarizes current evidence on sex-specific differences in epidemiology, pathophysiology, clinical presentation, prognosis, and treatment in women with AF and HF.

**Recent Findings:**

Emerging evidence indicates that women with AF and HF constitute a distinct clinical and biological phenotype. Women typically develop AF at older age, more commonly present with hypertension and HF with preserved ejection fraction (HFpEF), and report greater symptom burden and impaired quality of life. Sex-specific mechanisms including hormonal influences, atrial fibrosis, endothelial dysfunction, inflammation, autonomic dysregulation, and microvascular disease contribute to these differences. Population studies further demonstrate sex-related disparities in HF development and outcomes across AF subtypes.

**Summary:**

Despite increasing awareness, women remain underrepresented in clinical trials and are less likely to receive rhythm-control strategies, catheter ablation, or device therapy. Recognition of the “female fingerprint” in AF and HF is essential for individualized risk stratification and the advancement of precision cardiovascular medicine.

**Graphical Abstract:**

**Figure:**
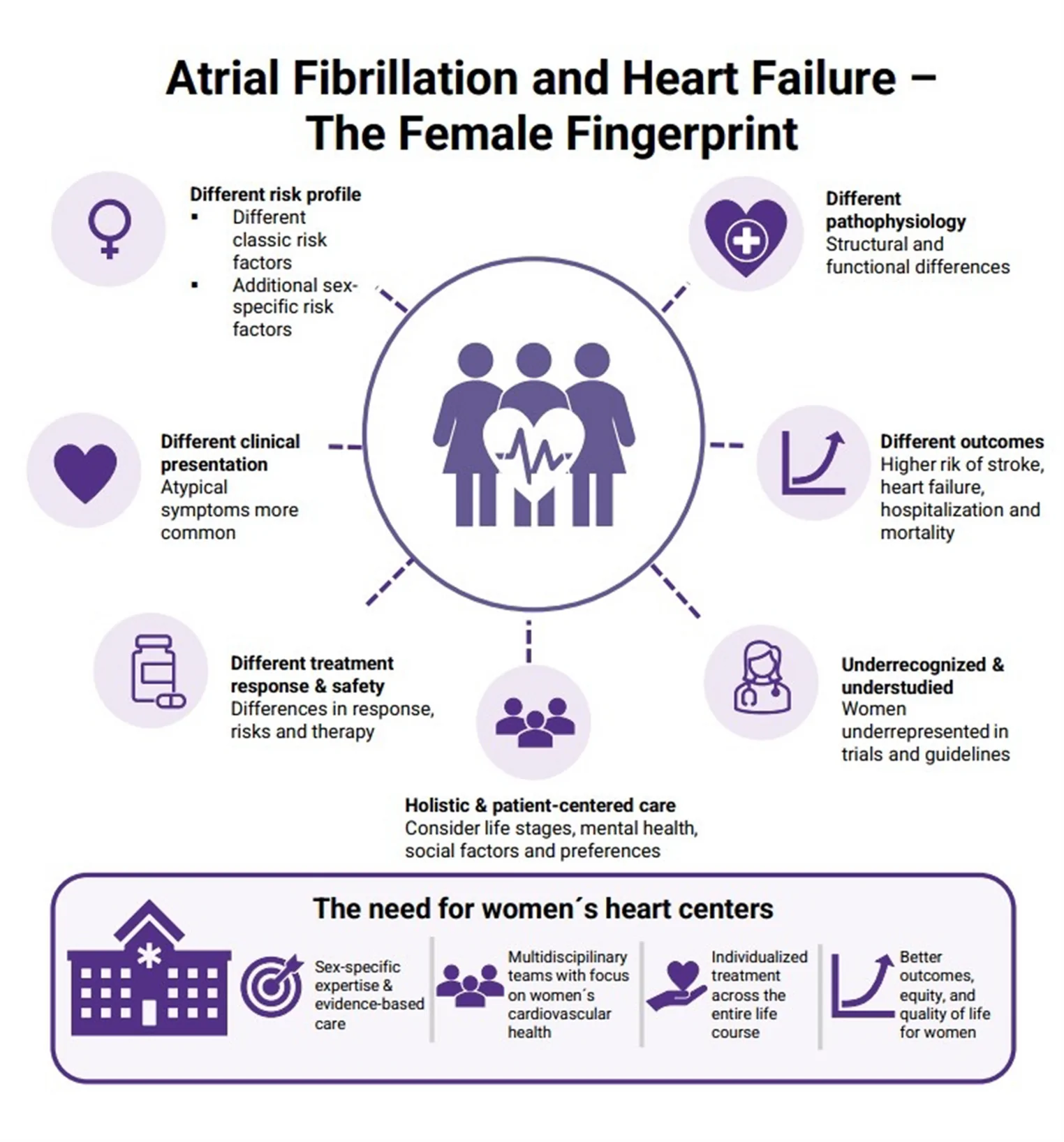

## Introduction

Atrial fibrillation (AF) and heart failure (HF) are highly prevalent cardiovascular disorders with increasing incidence attributable to demographic aging, improved survival from cardiovascular disease, and the growing burden of cardiometabolic risk factors. Both entities frequently coexist and interact through complex bidirectional mechanisms [1, 2]. AF contributes to HF progression through rapid ventricular response, irregular ventricular filling, loss of atrial systole, neurohormonal activation, and tachycardia-induced cardiomyopathy. Conversely, HF promotes atrial remodeling, fibrosis, elevated filling pressures, and electrophysiological alterations favoring AF initiation and maintenance [3].

The coexistence of AF and HF is associated with substantially increased risks of stroke, hospitalization, impaired functional capacity, reduced quality of life, and mortality. Importantly, accumulating evidence indicates that sex significantly influences disease development, progression, and therapeutic outcomes in both AF and HF [1, 4, 5].

Historically, cardiovascular disease has been conceptualized predominantly from a male perspective because women were substantially underrepresented in landmark cardiovascular trials [6–8]. Consequently, sex-specific manifestations of AF and HF remained underrecognized for decades. Contemporary studies now demonstrate that women exhibit a distinct cardiovascular phenotype characterized by:

- predominance of HF with preserved ejection fraction (HFpEF),
- increased frailty at time of diagnosis of HF and AF.
- higher symptom burden,
- different common and female-specific risk factors,
- and differential responses to therapeutic interventions.

The concept of a “female fingerprint” has emerged to describe the unique interaction of genetic, hormonal, structural, sociocultural and psychosocial determinants shaping cardiovascular disease expression in women. Recognition of these differences is increasingly important in the era of precision medicine and individualized cardiovascular care.

This review aims to provide a comprehensive overview of current evidence regarding sex-specific aspects of AF and HF, with emphasis on epidemiology, pathophysiological mechanisms, clinical presentation, diagnostic challenges, therapeutic implications, and future perspectives.

## Epidemiology

### Global Burden of AF and HF in Women

AF is the most common sustained cardiac arrhythmia worldwide, affecting more than 60 million individuals globally [9]. Also, HF affects over 60 million people and remains one of the leading causes of hospitalization and cardiovascular mortality [10–12]. Although AF incidence is generally higher in men, women account for approximately half of the overall AF population due to their longer life expectancy [9]. HF and AF often coexist, resulting in poor cardiovascular outcome. It is know, that in patients with preexisting AF, the development of HF led to rates of mortality three times higher in both men and women. Viceverda, in HF patients, the development of AF leads to a significant increase in mortality in women when compared to men [13, 14].

Women typically develop AF 5–10 years later than men and often present with a greater burden of nonischemic cardiovascular comorbidities including arterial hypertension and valvular heart disease [15–17].

In contrast to men, women predominantly develop HFpEF – and this applies particularly to women with preexisting AF [12] - whereas HFrEF is more commonly associated with male sex and ischemic cardiomyopathy [5, 18]. This distinction is clinically important because AF is particularly prevalent in HFpEF and significantly contributes to exercise intolerance, elevated filling pressures, and recurrent hospitalization.

### Sex Differences in AF Subtypes and HF Development

Recent population-based investigations have provided important insights into sex-specific trajectories of AF-related HF development. The Tromsø Study demonstrated that HF risk varies substantially according to AF subtype and biological sex. Women with permanent AF exhibited particularly high rates of incident HF, whereas men more frequently developed HF in association with persistent AF and underlying structural heart disease [19]. These findings suggest distinct mechanistic pathways:

- women appear more vulnerable to chronic pressure overload, diastolic dysfunction, and atrial-ventricular coupling abnormalities,
- whereas men more frequently develop HF due to ischemic remodeling and systolic dysfunction.

Moreover, Espnes et al. demonstrated that hypertension, obesity, and aging were particularly strong predictors of HF in women with AF, whereas coronary artery disease and systolic dysfunction predominated in men [2]. These observations reinforce the concept that AF-HF syndromes differ fundamentally between sexes.

## Pathophysiological Mechanisms

The pathophysiology of heart failure (HF) and atrial fibrillation (AF) involves complex structural, genetic, and physiological cardiac changes. Sex hormones and sex-specific biological mechanisms substantially influence the incidence and progression of both conditions.

### Hormonal Modulation

Sex hormones significantly affect atrial remodeling and arrhythmogenesis. Estrogen and progesterone influence autonomic nervous system activity, ion channel function, imflammation and atrial fibrosis. [20, 21]. Premenopausal estrogen exposure appears to provide partial protection against adverse myocardial remodeling through antifibrotic and anti-inflammatory mechanisms. Following menopause, declining estrogen levels contribute to: arterial stiffening, endothelial dysfunction, oxidative stress, increased collagen deposition, and myocardial fibrosis. These alterations favor both AF initiation and HFpEF development. Interestingly, women often demonstrate more extensive atrial fibrosis despite smaller left atrial size compared with men [22, 23]. Cardiac magnetic resonance imaging studies using late gadolinium enhancement consistently demonstrate higher fibrotic burden in women with AF [24].

### Atrial Remodeling and Fibrosis

Atrial fibrosis represents a key mechanistic link between AF and HF. Structural remodeling is driven by neurohormonal activation, inflammation, oxidative stress, and chronically elevated atrial pressure. Recent cardiac magnetic resonance imaging studies and data from invasive EP mapping procedures demonstrate that women with AF exhibit greater diffuse atrial fibrosis, enhanced atrial stiffness, lower atrial voltage, and more pronounced electromechanical dissociation despite smaller left atrial dimensions compared with men [22, 24]. Increased fibrotic burden has been associated with more severe symptoms, impaired exercise capacity, elevated left atrial pressure, lower catheter ablation efficacy, and increased susceptibility to HFpEF.

### Ventricular Remodeling and HFpEF

HFpEF is the predominant HF phenotype in women and is strongly associated with AF [12]. Contemporary evidence indicates that women develop concentric left ventricular remodeling, impaired diastolic relaxation, myocardial stiffness, endothelial dysfunction, and coronary microvascular disease more frequently than men [25, 26]. AF further aggravates HFpEF through loss of atrial contraction, irregular ventricular rhythm, tachycardia-mediated dysfunction, and elevated filling pressures. This interaction appears particularly relevant in elderly women with hypertension, obesity, and metabolic syndrome.

### Vascular Aging, Inflammation, and Autonomic Dysregulation

Accelerated vascular aging after menopause is considered central to the female AF–HF phenotype. Women exhibit greater increases in arterial stiffness, pulse pressure, and endothelial dysfunction, promoting left ventricular hypertrophy, atrial overload, and myocardial fibrosis. Inflammatory activation—enhanced by obesity, visceral adiposity, autoimmune disorders, and metabolic dysfunction—further contributes to oxidative stress, electrical remodeling, and AF maintenance [12, 13]. In addition, sex-related differences in autonomic regulation may alter atrial refractoriness and conduction heterogeneity, thereby increasing arrhythmogenic susceptibility in women.

## Clinical Presentation and Diagnostic Considerations

Women with AF and HF frequently present with a distinct clinical phenotype characterized by greater symptom burden, higher frailty prevalence, increased thromboembolic risk, and important diagnostic challenges compared with men [5, 13, 27, 28].

### Symptom Burden and Functional Impairment

Women with AF and HF consistently report more severe symptoms and poorer quality of life than men. Common clinical manifestations include: palpitations, dyspnea, fatigue, exercise intolerance, dizziness, anxiety and sleep disturbances [29, 30]. Notably, symptom severity in women does not always correlate with the degree of structural cardiac disease, suggesting important contributions from autonomic dysfunction, diastolic abnormalities, microvascular dysfunction, and psychosocial factors. Frailty is particularly common among elderly women with AF and HF and contributes substantially to adverse clinical outcomes, including: reduced mobility, recurrent hospitalization, polypharmacy, impaired treatment tolerance, cognitive decline, and loss of functional independence. Women additionally exhibit higher rates of depression and anxiety, which further influence symptom perception, healthcare utilization, and adherence to therapy [31, 32].

### Stroke Risk

Women with AF and concomitant HF are at particularly high risk for thromboembolic events and ischemic stroke [33]. Both conditions frequently coexist and share several pathophysiological mechanisms that promote thrombogenesis. In atrial fibrillation, ineffective atrial contraction and blood stasis—especially within the left atrial appendage—facilitate thrombus formation. Heart failure further contributes to this process through impaired cardiac output, endothelial dysfunction, systemic inflammation, and activation of neurohormonal pathways. Together, these mechanisms significantly increase the likelihood of thromboembolic complications [16, 34].

Several clinical studies have demonstrated that female patients with AF have a higher risk of stroke compared with male patients, particularly in the presence of additional cardiovascular risk factors such as advanced age, hypertension, diabetes mellitus, or heart failure [35, 36]. Consequently, female sex has historically been incorporated into the CHA₂DS₂-VASc score for stroke risk stratification [37, 38]. More recent evidence suggests that female sex should rather be considered a risk modifier than an independent risk factor; however, women with AF still experience higher rates of severe stroke and mortality.

A possible explanation for the increased rate of thromboembolic events and stroke in women with AF and HF may be the undertreatment or underdosing of oral anticoagulation (OAC). Several studies suggest that female patients are less likely to receive guideline-recommended anticoagulation therapy or may receive reduced dosages without clear clinical indications. In particular, inappropriate dose reduction of direct oral anticoagulants (DOACs) can lead to insufficient thromboembolic protection while not significantly decreasing bleeding risk [39, 40]. In women with concomitant HF, this issue may be even more relevant due to advanced age, renal impairment, or concerns regarding bleeding complications. Consequently, suboptimal anticoagulation management could contribute to the higher incidence of stroke and systemic embolism observed in female patients with AF.

In patients with both AF and HF, accurate risk assessment and timely initiation of oral anticoagulation are therefore essential to reduce stroke incidence and improve clinical outcomes. Current guidelines recommend individualized anticoagulation therapy based on thromboembolic risk evaluation and bleeding risk assessment [29].

### Diagnostic Challenges

Cardiovascular disease in women remains underrecognized and undertreated. The presence of atypical symptoms sometimes delays the diagnosis of AF and also HF, and consequently, the early initiation of treatment. Moreover, this often results in delayed presentation at more advanced stages of disease with decreased therapeutic options and outcomes [6, 40].

Various tools are used to diagnose and monitor the progression of atrial fibrillation and heart failure. The main prognostic biomarker is NT-proBNP, whose elevated levels decrease with age and are more common in women. This may be releated to hormonal influences, particularly testosterone-dependent regulation of neprilysin activity, which can reduce NT-proBNP levels. Women generally exhibit higher circulating natriuretic peptide concentrations than men for comparable HF severity [41]. Sex-specific interpretation of natriuretic peptide thresholds may therefore improve diagnostic accuracy and risk stratification.

Transthoracic echocardiography studies show that women with HFpEF generally have a higher ejection fraction, smaller left ventricular volumes, and less LV dilation than men, while LV hypertrophy remains comparable [42]. Left atrial volume index and pulmonary artery systolic pressure appear similar between sexes. Diastolic dysfunction is common in both men and women with HF and AF. However, some studies report higher E/e’ ratios and increased filling pressure in women, suggesting gearter LV stiffness, especially in HFpEF [12, 43]. Traditional echocardiographic parameters may therefore underestimate disease severity in women, particularly in HFpEF. Advanced imaging techniques, including strain echocardiography and cardiac magnetic resonance imaging (MRI), provide improved characterization of atrial dysfunction, diffuse myocardial fibrosis ventricular mechanics and coronary microvascular disease.

### Arrhythmia-Induced Cardiomyopathy

Arrhythmia-induced cardiomyopathy (AIC) describes left ventricular (LV) systolic dysfunction that is caused or aggravated by a sustained arrhythmia - most frequently AF - and that is partially or completely reversible once sinus rhythm or adequate rate control is restored [44]. Persistent rapid and irregular ventricular activation impairs calcium handling, promotes oxidative stress and neurohormonal activation, induces ventricular dyssynchrony, and ultimately drives myocyte remodeling and progressive chamber dilatation [44, 45]. Because recovery of LV function after restoration of sinus rhythm remains the diagnostic hallmark, AIC is by definition confirmed retrospectively. Distinguishing AF as the cause from AF as the consequence of HF therefore represents one of the central diagnostic challenges in this population.

This dependence on rhythm control for diagnosis has particular relevance for women. Women with AF are generally older and more often have hypertension and heart failure with preserved ejection fraction, and they are referred later and less frequently for cardioversion and catheter ablation [36]. Where rhythm control is withheld, the potentially reversible arrhythmic component of ventricular dysfunction may remain undetected, so that under-use of rhythm control translates directly into under-recognition of AIC.

Direct sex-specific data on AIC remain scarce. In the CAMERA-MRI trial, restoration of sinus rhythm by catheter ablation in patients with persistent AF and systolic dysfunction produced substantially greater LVEF improvement than rate control, and the absence of ventricular late gadolinium enhancement predicted greater recovery [46]. Given that women exhibit greater diffuse atrial fibrosis despite smaller atrial dimensions, it is plausible that fibrotic burden modifies the reversibility of ventricular dysfunction in a sex-dependent manner. This hypothesis has not yet been formally tested, and women were underrepresented in the trials from which the current understanding of AIC is derived (Table 1).

**Table 1:** Randomized trials of rhythm control and catheter ablation in patients with atrial fibrillation and heart failure: female representation

Trial (year)	Population	*n* (% women)	Principal findings	Sex-specific data
AATAC (2016)	Persistent AF, NYHA II–III, LVEF < = 40%, ICD/CRT-D	203 (26%)	AF-free survival 70% vs. 34% with amiodarone; lower mortality (8% vs. 18%) and hospitalization (31% vs. 57%)	Sex assessed; no effect of sex on AF recurrence
CAMERA-MRI (2017)	Persistent AF, LVEF < = 45%, idiopathic cardiomyopathy	68 (9%)	LVEF improvement + 18% vs. + 4.4%; normalization to > = 50% in 58% vs. 9%; LGE predicted recovery	Not reported; only 6 women enrolled
CASTLE-AF (2018)	Paroxysmal/persistent AF, NYHA II-IV, LVEF < = 35%, ICD/CRT-D	363 (14%)	Composite of all-cause mortality or HF hospitalization 28% vs. 44%; LVEF + 8% vs. 0.2%	Subgroup analysis: HR 0.93 (women) vs. 0.58 (men), interaction *p* = 0.36; limited by 13–16% female enrolment
CABANA (2019); sex analysis (2021)	AF, age > = 65 y or < 65 y with > = 1 stroke risk factor	2204 (37%)	No significant reduction of primary composite by intention-to-treat; reduced AF recurrence; benefit in HF subgroup	Primary outcome HR 1.01 (0.62–1.65) women vs. 0.73 (0.51–1.05) men, interaction *p* = 0.299; AF recurrence HR 0.64 vs. 0.48, interaction *p* = 0.060; fewer ancillary lesion sets in women (55.7% vs. 62.2%)
AMICA (2019)	Persistent AF, LVEF < = 35%, ICD/CRT-D	140 (10%)	No significant LVEF improvement with ablation	Sex-stratified outcomes not reported
EAST-AFNET 4 (2020); sex analysis (2023)	AF diagnosed < = 12 months with cardiovascular conditions	2789 (46%)	Early rhythm control reduced adverse cardiovascular outcomes	Women derived benefit comparable to men despite older age and higher symptom burden
CASTLE-HTx (2023); sex analysis (2025)	Symptomatic AF and end-stage HF eligible for heart transplantation	194 (19%)	Death, LVAD implantation or urgent transplantation 8.2% vs. 29.9%; HR 0.24 (0.11–0.52)	No treatment-by-sex interaction (*p* = 0.71); 1-year primary endpoint HR 0.33 (0.07–1.50) in women vs. 0.23 (0.09–0.59) in men; absolute benefit numerically larger in women (IR difference 21.4 vs. 13.6 per 100 patient-years)

AF, atrial fibrillation; CRT-D, cardiac resynchronization therapy defibrillator; HF, heart failure; HFrEF, heart failure with reduced ejection fraction; HR, hazard ratio; ICD, implantable cardioverter defibrillator; LGE, late gadolinium enhancement; LVAD, left ventricular assist device; LVEF, left ventricular ejection fraction; NYHA, New York Heart Association

## Gender-sensitive therapeutic management of patients with HF and AF

### Pharmacological Therapy

Current HF guidelines do not distinguish between men and women, despite growing evidence of sex-specific differences in drug efficacy and safety [47]. Women often require lower drug doses because of differences in metabolism, body composition, and hormonal status, and they experience adverse drug reactions more frequently [48, 49].

Standard HF therapy includes ACE inhibitors (ACEIs) or angiotensin receptor blockers (ARBs), beta-blockers, mineralocorticoid receptor antagonists (MRAs), angiotensin receptor-neprilysin inhibitors (ARNIs), and sodium-glucose cotransporter 2 inhibitors (SGLT2i). Studies suggest that women may achieve similar or greater benefits with lower doses of ACEIs, ARBs, and beta-blockers, whereas guideline target doses appear more effective in men [47, 50]. ARBs and beta-blockers have shown particularly favorable outcomes in women with HFrEF, partly due to lower adverse effect rates [51].

MRAs and ARNIs may also provide greater benefits in women, especially in HFpEF and HFmrEF. Trials such as TOPCAT and PARAGON-HF reported lower mortality and hospitalization rates in women treated with spironolactone or sacubitril/valsartan [51, 52]. Women with HFpEF may respond particularly well to ARNIs because of differences in neprilysin activity, NT-proBNP levels, and microvascular inflammation [51].

SGLT2i improve outcomes in both sexes, although some studies indicate greater reductions in worsening HF and cardiovascular death in women with HFrEF [47]. Despite a higher rate of urinary and genital infections in women, efficacy and overall safety remain comparable between sexes [53]. Emerging evidence also suggests sex-specific cardiometabolic effects of SGLT2i on myocardial metabolism, oxidative stress, and endothelial function [53].

### Rate vs. Rhythm Control in female patients with AF and HF

The AFFIRM and RACE trials found no significant difference in cardiovascular mortality between rate and rhythm control strategies in patients with HF [54, 55]. However, women showed a higher stroke risk and more thromboembolic events with rhythm control, as well as more adverse effects from antiarrhythmic drugs [54, 55].

In the era of non-vitamin K antagonist oral anticoagulants (NOACs) and modern catheter ablation techniques, rhythm control strategies have achieved significantly improved outcomes in patients with AF, and especially those with concomitant HF [56]. Evidence from contemporary trials such as the EAST-AFNET 4 study demonstrated that early rhythm-control therapy is associated with improved cardiovascular outcomes in both women and men [57]. Importantly, women appear to benefit from early rhythm-control strategies to a similar extent as male patients [58]. Nevertheless, female patients are still less likely to be referred for rhythm-control interventions in a timely manner. Several registries and observational studies indicate that women undergo cardioversion and catheter ablation less frequently and often at more advanced stages of disease progression [59]. This delayed referral pattern may contribute to worse symptom burden, lower quality of life, and potentially poorer cardiovascular outcomes in women with AF.

### Device Therapy

The rates of implantable cardioverter defibrilllator (ICD) implantations are similar in both sexes. Thus, women have higher complication rates after ICD implantation, including pneumothorax, hemorrhage, and lead dislodgement [60]. However, women often benefit more from CRT and CRT-D, showing greater improvements in quality of life, hospitalization rates, cardiac remodeling, and survival [61]. These benefits may be related to lower rates of HF and smaller heart dimensions in women. These observations support more individualized approaches to device selection. Current guidelines still lack sex-specific recommendations for device implantation thresholds [47, 62].

### Atrial Fibrillation in Advanced and End-Stage Heart Failure

The evidence supporting rhythm control in AF and HF is strongest at the severe end of the disease spectrum, yet this is where women are least represented (Table 1). In CASTLE-AF, catheter ablation in patients with AF and LVEF < = 35% reduced the composite of all-cause mortality and HF hospitalization (28% versus 44%) and improved LVEF compared with conventional rate or rhythm control [63]. Only 52 of 363 randomized patients (14%) were women. A subgroup analysis found no statistically significant sex difference in the primary endpoint, although the numerical trend favored men and the analysis was limited by female enrollment of 13–16% per treatment arm [64]. Across the randomized trials summarized in Table 1, female participation ranges from 9% to 26%, and only two trials assessed sex as an outcome modifier [64].

CASTLE-HTx extended this evidence to end-stage disease. Among 194 patients with symptomatic AF and end-stage HF who were eligible for heart transplantation, ablation reduced the composite of death, LVAD implantation or urgent transplantation from 29.9% to 8.2% [65]. A dedicated sex-specific analysis has since been published: among 37 women and 157 men, no interaction between sex and treatment was observed for the primary endpoint, all-cause death, change in LVEF, or reduction in AF burden [66].

Women were considerably better represented in CABANA, which randomized 819 women (37%) and 1385 men [67]. Compared with men, women were older, more symptomatic, and more frequently had symptomatic HF. In the prespecified sex analysis, no clinically relevant treatment-by-sex interaction was observed for the primary composite outcome. AF recurrence was reduced by ablation in both sexes, although the effect was numerically greater in men. Women were less likely to undergo ancillary non-pulmonary vein ablation during the index procedure, and adverse events did not differ by sex [68]. In the CABANA heart failure subgroup, ablation was associated with improved survival and greater freedom from AF [69].

Taken together, these trials support rhythm control across the entire spectrum of HF severity, including patients awaiting transplantation, without evidence that women derive less clinical benefit than men. The persistent under-enrollment of women, however, means that confidence intervals around female-specific estimates remain wide and that no trial in advanced HF has been powered for a sex-stratified endpoint. The problem is not limited to trials: in clinical practice, women with AF and HF are about half as likely as men to undergo catheter ablation [70].

## Conclusion

This review discusses the close relationship between AF and HF, two common cardiovascular diseases that frequently coexist and significantly worsen prognosis. The article highlights that women show important sex-specific differences in the development, presentation, and treatment response of AF and HF. Women are more likely to develop HFpEF, experience a higher symptom burden, greater frailty, and an increased risk of stroke and hospitalization compared with men.

Hormonal changes after menopause, increased atrial fibrosis, vascular aging, inflammation, and endothelial dysfunction contribute to the distinct female cardiovascular phenotype. In addition, women often present with atypical symptoms, leading to delayed diagnosis and treatment. Despite evidence that women benefit from modern HF therapies and early rhythm-control strategies, they remain underrepresented in clinical trials and are less frequently referred for advanced interventions such as catheter ablation.

### Future Perspectives and Precision Medicine

The review underlines the importance of recognizing the “female fingerprint” in cardiovascular disease and integrating sex-specific factors into clinical decision-making. In this context, specialized women’s heart centers are essential to ensure optimal cardiovascular care for women. These centers can improve early diagnosis, provide individualized and multidisciplinary treatment, and address female-specific risk factors such as menopause, pregnancy-related complications, and psychosocial aspects. Women’s heart centers therefore represent an important step toward more precise, equitable, and personalized cardiovascular medicine.

## Key References

- Rivera FB, Magalong JV, Bantayan NRB et al. Participation of Women in Cardiovascular Trials From 2017 to 2023: A Systematic Review. JAMA Netw Open 2025;8:e2529104.
  - ○ Important article highlighting the underrepresentation of women in cardiovascular trials.
- Espnes H, Wilsgaard T, Ball J et al. Heart Failure in Atrial Fibrillation Subtypes in Women and Men in the Tromso Study. JACC Adv 2025;4:101556.
  - ○ A highly relevant and timely, randomized study investigating HF and AF subtypes and their impact on outcomes in women and men.
- Nakashima K, Yamaguchi T, Takahashi Y et al. Sex differences in atrial fibrillation-related atrial remodelling assessed by electroanatomic mapping and biopsy. Eur Heart J 2026;47:217–231.
  - ○ Important novel study investigating the pathophysiological differences in atrial remodeling between women an men.

## Data Availability

No datasets were generated or analysed during the current study.
